# Work-life balance as a mediator between perceived stress and work withdrawal behavior among critical care nurses in China: a multicenter cross-sectional study

**DOI:** 10.3389/fpubh.2026.1776976

**Published:** 2026-05-08

**Authors:** Jun Li, Jun Wang, Shuai Li, Dabing Dai, Yu Xu

**Affiliations:** 1 Department of Outpatient, West China Hospital, Sichuan University/West China School of Nursing, Sichuan University, Chengdu, China; 2 Department of Emergency Medicine, General Hospital of Western Theater Command, Chengdu, China; 3 Department of Critical Care Medicine, West China Hospital of Sichuan University, Chengdu, China

**Keywords:** Intensive Care Unit, mediating effect, nurses, perceived stress, work withdrawal behavior, work-life balance

## Abstract

**Background:**

The working environment of ICU nurses, which is high-intensity and fast-paced, contributes to elevated levels of perceived stress, which may consequently lead to work withdrawal behavior. Although work-life balance is regarded as a critical resource in coping with perceived stress, its mediating mechanism between perceived stress and work withdrawal behavior has not yet been fully elucidated.

**Objective:**

This study aims to examine the relationships between perceived stress, work-life balance, and work withdrawal behavior among ICU nurses in China.

**Methods:**

This cross-sectional study was conducted between September and October 2025. A total of 1,279 ICU nurses were enrolled from 58 hospitals across 16 Chinese provinces using convenience and snowball sampling. Data were collected employing validated scales: the Work-Life Balance Scale (WLBS), the Perceived Stress Scale (PSS), and the Work Withdrawal Behavior Scale (WWBS). Structural equation modeling (SEM) was applied to examine the relationships among variables, and the bootstrap method was utilized to test the mediating effects.

**Results:**

The questionnaire response rate was 91.35% (1,279/1400). Path analysis confirmed that perceived stress negatively influenced work-life balance (*β* = −0.234, *p* < 0.001) and positively influenced work withdrawal behavior (*β* = 0.207, *p* < 0.001). Work-life balance also showed a significant negative effect on withdrawal behavior (*β* = −0.439, *p* < 0.001). The mediation analysis indicated that work-life balance played a significant mediating role between perceived stress and work withdrawal behavior, with the indirect effect accounting for 33.23% of the total effect.

**Conclusion:**

The results indicate a significant association between perceived stress and work withdrawal behavior, with work-life balance playing a crucial mediating role in this relationship. These findings highlight the potential of work-life balance-focused interventions to alleviate stress, reduce withdrawal, and improve occupational health outcomes for ICU nurses.

## Introduction

The working environment of Intensive Care Unit (ICU) nurses is characterized by high-intensity, rapid-paced, and highly stressful conditions, which frequently leads to significant perceived stress among nurses (1–3). Existing research has largely confirmed the association between such stress and work withdrawal behavior (3, 4). According to the Conservation of Resources (COR) theory, individuals strive to obtain, retain, protect, and foster personal resources to cope with threats of potential or actual resource loss (5). In the resource-depleting context of the ICU, if nurses cannot effectively replenish or conserve their resources, they become more susceptible to job burnout, heightened perceived stress, and work withdrawal behavior (6).

Perceived stress, particularly when assessed using the Perceived Stress Scale-10, is widely recognized as a key indicator of job burnout and mental health issues (7). Due to frequent exposure to critically ill patients, life-and-death situations, and significant care responsibilities, ICU nurses generally report higher levels of perceived stress compared to nurses in other departments (1–3, 8). This elevated perceived stress not only adversely affects nurses’ psychological wellbeing but may also contribute to the accumulation of negative emotions (9, 10), such as anger, which can subsequently impair work engagement and perceptions of organizational support (11). At the same time, factors such as poor relationships with colleagues, poorly organized work, low perceived job control, limited influence over work schedules, and high job demands can all contribute to an increase in perceived stress (12, 13).

Work withdrawal behavior represents a maladaptive response to work-related stress, characterized by reduced engagement, active disengagement from tasks, and ultimately turnover (4, 14). Among ICU nurses, such behavior may manifest as absenteeism, tardiness, decreased efficiency, diminished empathy toward patients, and increased turnover intention (4). High-intensity workloads are consistently linked to increased job burnout and greater work-to-family conflict (15). When nurses experience excessive perceived stress, they may reduce their psychological or physical presence at work as a strategy to conserve remaining personal resources—a perspective consistent with the Conservation of Resources theory (4, 16). For instance, workplace ostracism has been shown to lower job satisfaction and performance, increase turnover intention, and contribute to emotional exhaustion (4).

Work-life balance is regarded as an important personal resource, reflecting an individual’s perceived satisfaction with the equilibrium between work and non-work domains (17, 18). For ICU nurses, achieving work-life balance is particularly challenging due to shift work, overtime, and irregular schedules (18, 19). Family-friendly work practices and supportive supervisory behaviors have been shown to effectively reduce work–family conflict, enhance job satisfaction, and lower turnover intention (15, 20). However, in low-trust management environments, even increased family-friendly practices may fail to alleviate perceived stress and can, in some cases, exacerbate it (21). This suggests that attaining work-life balance depends not only on organizational policies but also on employees’ trust in management and a supportive organizational culture (21).

Existing studies have shown a negative correlation between perceived stress and work-life balance (10, 20); that is, higher perceived stress is often associated with lower levels of work-life balance. According to the COR theory, work-life balance itself can be regarded as a critical personal resource that helps individuals resist resource loss and promote resource gain (5, 22). When work-life balance is impaired, ICU nurses face a net loss of resources (e.g., time, energy, recovery opportunities), leaving them with insufficient psychological and temporal resources to cope with work-related stress, thereby increasing the likelihood of work withdrawal behavior (4, 23). Accordingly, within the framework of COR theory, work-life balance plays a core mediating role in the pathway from perceived stress to behavioral withdrawal: perceived stress → impaired work-life balance (resource loss) → insufficient coping resources → work withdrawal behavior (16). Improving work-life balance not only directly enhances employee wellbeing but also indirectly mitigates the negative impact of stress on organizational effectiveness by interrupting the resource loss spiral and promoting resource gain. This mediating pathway highlights the importance of dual interventions: organizational support (e.g., family-friendly policies and a trustworthy management environment) and individual strategies to maintain and promote work-life balance in high-intensity work settings.

Previous studies have confirmed that among nurses in general, perceived stress is negatively associated with work-life balance and positively associated with work withdrawal behavior (4). However, these relationships have not been directly verified in the specific context of ICU nurses—a high-stress, high-turnover population. Moreover, although the mediating logic based on COR theory is theoretically plausible, whether work-life balance actually mediates the effect of perceived stress on work withdrawal behavior among ICU nurses remains empirically untested. Therefore, grounded in COR theory, this study aims to explore the relationships among perceived stress, work-life balance, and work withdrawal behavior in ICU nurses and proposes the following hypotheses:

*Hypothesis 1*: Perceived stress is significantly positively correlated with work withdrawal behavior among ICU nurses.

*Hypothesis 2*: Work-life balance is significantly negatively correlated with work withdrawal behavior among ICU nurses.

*Hypothesis 3*: Work-life balance partially mediates the relationship between perceived stress and work withdrawal behavior among ICU nurses.

The findings of this study will contribute to a deeper understanding, from the perspective of COR theory, of the mechanisms underlying work withdrawal behavior in ICU nurses, and will provide both theoretical and empirical foundations for developing effective interventions to improve nurses’ occupational health and wellbeing (4). By promoting work-life balance, it may be possible not only to alleviate nurses’ perceived stress but also to effectively reduce the incidence of work withdrawal behavior, thereby enhancing the quality and stability of healthcare services.

## Methods

### Study design and participants

This study employed a multicenter cross-sectional design. Between September and October 2025, a convenience and snowball sampling approach was used to recruit 1,279 ICU nurses from 58 primary, secondary, and tertiary hospitals across 16 provinces in China. The study adhered strictly to the Strengthening the Reporting of Observational Studies in Epidemiology (STROBE) statement to ensure transparency and completeness (24). Inclusion criteria were: (1) possession of a valid nurse practicing certificate and official registration; (2) full-time employment status under a formal labor contract or within the hospital’s permanent staffing system; (3) current clinical assignment in an ICU; (4) voluntary participation with written informed consent; and (5) employment in a primary, secondary, or tertiary hospital. Exclusion criteria included: (1) temporary absence due to illness, personal leave, or external training; (2) current enrollment in educational programs, internships, or formal training; (3) primary responsibilities in teaching or research with limited engagement in clinical practice; and (4) responses submitted within 180 s, those generated from non-mobile devices, and data containing errors.

### Sample size

The required sample size was determined based on the complexity of the structural equation model (SEM). The overall model involved 99 free parameters. Following established guidelines (25, 26), a minimum ratio of 5–10 cases per free parameter is recommended for reliable estimation using maximum likelihood. Thus, at least 495–990 participants were required. To account for potential invalid responses, we distributed 1,400 questionnaires. After excluding incomplete or careless responses, 1,279 valid cases were retained (effective response rate: 91.4%). The final sample provided a case to parameter ratio of approximately 12.9:1, which exceeds the recommended thresholds and ensures adequate statistical power for model testing (27).

### Data collection

Data collection was conducted using electronic questionnaires administered via the Questionnaire Star online platform. Participants first reviewed and submitted an electronic informed consent form before proceeding to complete the questionnaire. The preface of the questionnaire included detailed instructions outlining the study’s purpose, methodology, significance, and completion requirements, and explicitly stated that participation was voluntary, anonymous, and based on the nurses’ genuine perceptions. To ensure data completeness and accuracy, we excluded 23 questionnaires completed on non mobile devices due to the shared computer environment, and removed 30 questionnaires with patterned responses (e.g., straightlining). The questionnaire comprised 53 items. Following established guidelines for detecting insufficient effort responding (28), we adopted the “two seconds per item” rule as the theoretical lower bound for valid responses, yielding a minimum completion time of 106 s (53 items × 2 s/item). However, pilot test data (*n* = 50) showed that the 5th percentile of completion times was 175 s. Considering individual differences in reading speed and item complexity, we adopted a more conservative cutoff of 180 s. Ultimately, 68 questionnaires with completion times below 180 s were excluded.

### Ethics approval

This study was approved by the Institutional Biomedical Ethics Committee of the West China Hospital, Sichuan University (Approval No. 20242520). All participants were fully informed and provided electronic consent prior to the survey. All data were anonymized before analysis to protect participant privacy.

### Measurement tools

#### General characteristics questionnaire

Based on the COR theory and expert panel discussions, the demographic variables, comprising a total of 14 items, were categorized into three domains (29, 30): Personal Resources: service years, age, gender, marital status, educational background, ICU work duration, professional title, and work position. Material Resources: hospital nature, hospital level, and department. Social Resources: monthly income, night shift work in the past year, and presence of any chronic diseases.

#### Work-Life Balance Scale

The Work-Life Balance Scale (31) comprises three dimensions: Time Balance (6 items), assessing the allocation of time between work and personal life; Involvement Balance (5 items), evaluating psychological engagement across domains; and Satisfaction Balance (6 items), measuring overall satisfaction with work and life. All items are rated on a 6-point Likert scale from 1 (Not at all) to 6 (Completely), with higher scores reflecting better work-life balance. While the original scale reported a Cronbach’s *α* of 0.86 (31), in this study the values were 0.948, 0.896, and 0.909 for the three dimensions, respectively, with an overall Cronbach’s *α* of 0.965. The standardized factor loadings of all items ranged from 0.585 to 0.923 (all *p* < 0.001). The composite reliabilities (CR) for time balance, involvement balance, and satisfaction balance were 0.945, 0.890, and 0.847, respectively, and the average variances extracted (AVE) were 0.743, 0.619, and 0.487, respectively. Although the AVE for satisfaction balance was slightly below 0.50, its CR reached 0.847 and all factor loadings were significant.

#### Perceived Stress Scale

The Perceived Stress Scale, originally developed by Zhen et al. (32), was adapted into Chinese by Wang et al. (33), who reported a Cronbach’s *α* of 0.91. The Chinese version comprises two dimensions: Negative Stress Perception (6 items), assessing negative emotional and physical responses to stress, and Positive Stress Perception (4 items), measuring adaptive coping and emotional regulation. All 10 items are rated on a 5-point Likert scale (1 = never to 5 = very often), with higher total scores indicating greater perceived stress. In the present study, the scale showed high internal consistency, with Cronbach’s *α* coefficients of 0.952 and 0.920 for the two dimensions, respectively, and 0.938 for the overall scale. The standardized factor loadings of all items ranged from 0.846 to 0.895 (all *p* < 0.001). The composite reliabilities (CR) for positive stress perception and negative stress perception were 0.920 and 0.952, respectively, and the average variances extracted (AVE) were 0.742 and 0.766, respectively.

#### The Work Withdrawal Behavior Scale

The Work Withdrawal Behavior Scale, originally developed by Lehman et al. (34) and adapted into Chinese by Tian et al. (35), was used to assess employees’ withdrawal behavior over the past year. The scale consists of two dimensions: Psychological Withdrawal (8 items), measuring emotional avoidance and cognitive disengagement in response to work difficulties, and Behavioral Withdrawal (4 items), assessing physical withdrawal due to work-related stress. Items are rated on a 5-point Likert scale from 1 (Never) to 5 (Always), with higher total scores indicating stronger withdrawal behavior. The Chinese version reported a Cronbach’s *α* of 0.926; in this study, values were 0.891 and 0.933 for the two dimensions, respectively, and 0.928 for the full scale. The standardized factor loadings of all items ranged from 0.474 to 0.917 (all *p* < 0.001). The composite reliabilities (CR) for behavioral withdrawal and psychological withdrawal were 0.935 and 0.900, respectively, and the average variances extracted (AVE) were 0.784 and 0.537, respectively.

### Statistical analysis

All analyses were conducted using SPSS 26.0 and AMOS 28.0. Categorical data are presented as frequencies (percentages). Normally distributed continuous data, as assessed by normality tests, are expressed as mean ± standard deviation. Group comparisons were performed using independent samples *t*-tests for two groups and one-way ANOVA for multiple groups, with the statistical significance level set at *p* < 0.05 (two-tailed). Bivariate associations among work-life balance, perceived stress, and work withdrawal behavior were examined using Pearson correlation analysis. Guided by the COR theory, a structural equation model (SEM) was constructed in AMOS 28.0 to examine the path relationships among variables. The model incorporated core individual and social resource variables. Covariates were included if they were significant in univariate analyses and showed no evidence of multicollinearity, as assessed by the variance inflation factor (VIF) (36). Mediation effects were tested using the bias-corrected nonparametric percentile bootstrap method with 5,000 resamples to estimate direct and indirect effects and their corresponding 95% confidence intervals.

## Results

A total of 1,400 questionnaires were distributed, of which 1,279 valid responses were collected, yielding a valid response rate of 91.35%.

### General characteristics of the participants

Participants were mainly recruited from state hospitals, tertiary hospitals, and general ICUs. The majority were frontline clinical ICU nurses who were female, held a bachelor’s degree, and worked night shifts. Regarding work withdrawal behavior scores, higher values were observed among nurses from private hospitals, those with senior professional titles, and those with chronic diseases. Significant differences in work withdrawal scores were found across several variables, including ICU work duration, professional title, age, service years, marital status, presence of chronic diseases, and monthly income (all *p* < 0.05; Table 1).

**Table 1:** General characteristics of the participants.

Variable	*n* (%)	Work withdrawal behavior (mean ± SD)	Statistic	*p*
Total	1,279 (100)	20.374 ± 8.235		
Hospital nature			*t* = −1.510	0.142
State hospital	1,249 (97.65)	20.287 ± 8.059		
Private hospital	30 (2.35)	24.000 ± 13.409		
Hospital level			*F* = 0.026	0.975
Tertiary hospital	1,134 (88.66)	20.368 ± 8.371		
Secondary hospital	134 (10.48)	20.463 ± 7.064		
Primary hospital	11 (0.86)	19.909 ± 7.943		
Department			*F* = 2.551	0.078
General ICU	1,110 (86.79)	20.570 ± 8.339		
Surgical ICU	91 (7.12)	19.407 ± 8.245		
Medical ICU	78 (6.10)	18.705 ± 6.335		
ICU work duration			*F* = 5.334	0.001
1–5	506 (39.56)	19.257 ± 7.837		
6–10	348 (27.21)	21.218 ± 8.001		
11–20	384 (30.02)	21.086 ± 8.694		
≥21	41 (3.21)	20.317 ± 9.248		
Position			*t* = −0.246	0.806
Clinical	1,135 (88.74)	20.359 ± 8.494		
Administrative	144 (11.26)	20.493 ± 5.829		
Professional title			*F* = 4.932	0.007
Primary	710 (55.51)	19.808 ± 8.457		
Intermediate	502 (39.25)	20.888 ± 7.921		
Senior	67 (5.24)	22.507 ± 7.640		
Education			*F* = 0.082	0.922
Associate degree and below	235 (18.37)	20.187 ± 9.109		
Bachelor’s degree	1,002 (78.34)	20.409 ± 8.075		
Master’s degree and above	42 (3.28)	20.571 ± 6.936		
Age			*F* = 6.770	0.001
20–29	476 (37.22)	19.294 ± 7.682		
30–39	678 (53.01)	21.091 ± 8.586		
≥40	125 (9.77)	20.592 ± 7.941		
Service years			*F* = 8.607	<0.001
1–5	407 (31.82)	18.985 ± 7.508		
6–10	334 (26.11)	20.949 ± 8.161		
≥11	538 (42.06)	21.067 ± 8.679		
Gender			*t* = 1.692	0.091
Male	217 (16.97)	21.235 ± 8.354		
Female	1,062 (83.03)	20.198 ± 8.203		
Marital status			*F* = 10.252	<0.001
Married	828 (64.74)	21.087 ± 8.676		
Unmarried	422 (33.00)	18.900 ± 7.071		
Divorced or widowed	29 (2.27)	21.448 ± 8.454		
Night shift			*t* = 1.373	0.170
No	162 (12.67)	21.204 ± 7.353		
yes	1,117 (87.33)	20.253 ± 8.351		
Monthly income			*F* = 3.211	0.022
≤5,000	259 (20.25)	20.336 ± 9.226		
5,001–8,000	539 (42.14)	20.781 ± 8.572		
8,001–11,000	287 (22.44)	20.749 ± 8.078		
>11,000	194 (15.17)	18.737 ± 5.512		
Presence of chronic disease			*t* = −3.734	<0.001
No	1,068 (83.50)	19.993 ± 7.934		
Yes	211 (16.50)	22.299 ± 9.403		

SD, standard deviation; *t*, *t*-tests; *F*, ANOVA; ICU, Intensive Care Unit.

### Scores on work-life balance, perceived stress, and work withdrawal behavior of the participants

The total score was 70.69 ± 18.74 for the Work-Life Balance Scale, 20.37 ± 8.24 for the Work Withdrawal Behavior Scale, and 33.03 ± 9.14 for the Perceived Stress Scale (Table 2).

**Table 2:** Scores on work-life balance, perceived stress, and work withdrawal behavior of the participants.

Variables	Score (mean ± SD)	Item score (mean ± SD)
Work -Life Balance Scale	70.69 ± 18.74	4.16 ± 1.10
Time balance	23.60 ± 7.33	3.93 ± 1.22
Involvement balance	21.48 ± 5.68	4.30 ± 1.14
Satisfaction balance	25.61 ± 6.96	4.27 ± 1.16
Work Withdrawal Behavior Scale	20.37 ± 8.24	1.70 ± 0.69
Psychological withdrawal	14.97 ± 5.84	1.87 ± 0.73
Behavioral withdrawal	5.40 ± 2.92	1.35 ± 0.73
Perceived Stress Scale	33.03 ± 9.14	3.30 ± 0.91
Negative Stress perception	19.77 ± 6.19	3.29 ± 1.03
Positive stress perception	13.27 ± 4.06	3.32 ± 1.02

SD, standard deviation.

### Correlation analysis of work-life balance, perceived stress, and work withdrawal behavior among the participants

In the present study, as shown in Table 3, a significant negative correlation was observed between perceived stress and work life balance (*r* = −0.203, *p* < 0.01). Perceived stress showed a significant positive correlation with work withdrawal behavior (*r* = 0.261, *p* < 0.01), while work life balance was significantly negatively correlated with work withdrawal behavior (*r* = −0.457, *p* < 0.01).

**Table 3:** Correlation analysis of work-life balance, perceived stress, and work withdrawal behavior among the participants.

Correlations	Perceived Stress Scale	Work Life Balance Scale	Work Withdrawal Behavior Scale
Perceived Stress Scale	1		
Work Life Balance Scale	−0.203**	1	
Work Withdrawal Behavior Scale	0.261**	−0.457**	1

**p<0.01.

### Structural equation modeling for perceived stress, work-life balance, and work withdrawal behavior among participants

To clarify the relationships among variables, factors identified as statistically significant in the univariate analysis—including professional title, age, service years, marital status, presence of chronic diseases, and monthly income—were included as control variables in a structural equation model (SEM). Path analysis using AMOS 28.0 was conducted to test the mediation model, with perceived stress as the independent variable, work-life balance as the mediator, and work withdrawal behavior as the dependent variable, to estimate direct and indirect effects.

**Figure 1:**
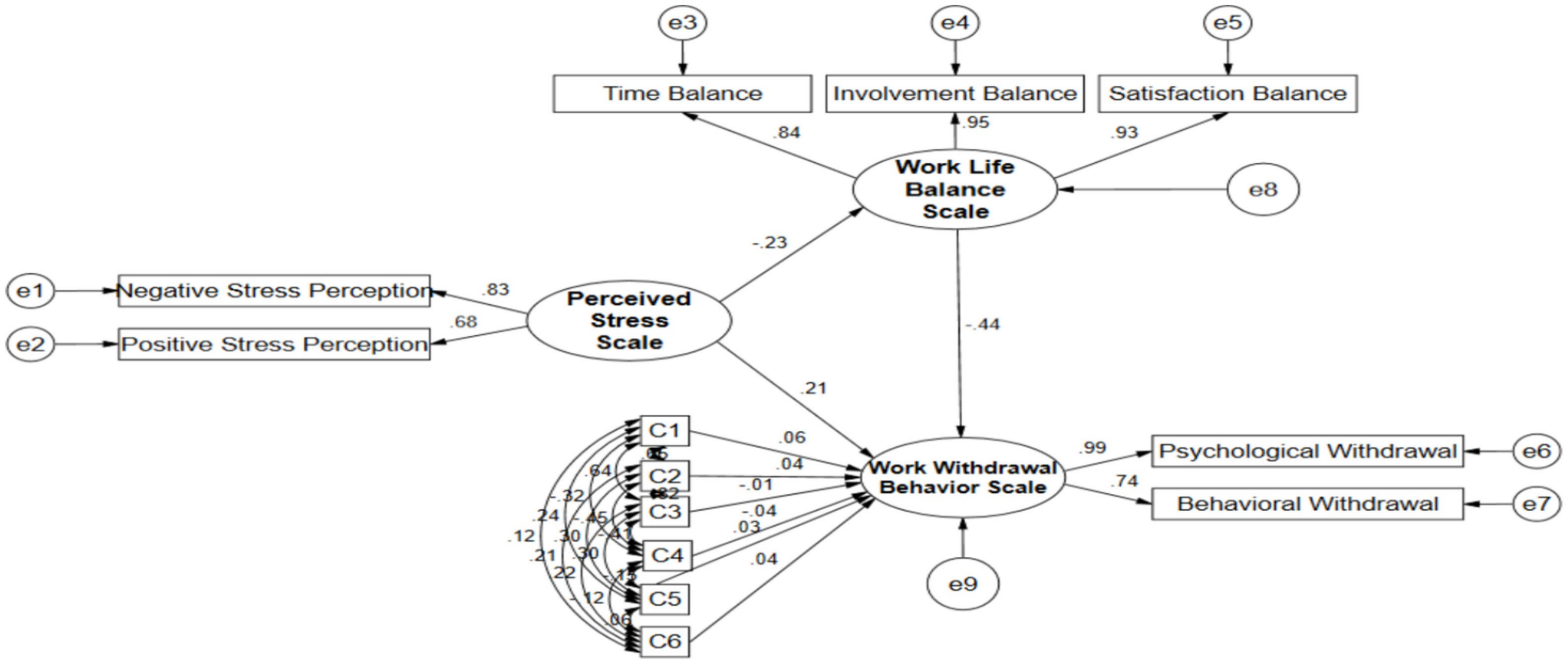
Structural equation modeling for perceived stress, work-life balance, and work withdrawal behavior among participants.

The model fit indices were as follows: *χ*^2^/df = 4.962; GFI = 0.972, NFI = 0.972, IFI = 0.977, CFI = 0.977, and TLI = 0.962, all exceeding 0.9, indicating a relatively high comparative fit of the model. The RMSEA was 0.056, which is below the 0.08 threshold, suggesting an acceptable approximate error and good model fit (37). The path analysis results (Figure 1) showed that perceived stress had a significant negative effect on work-life balance (*β* = −0.234, *p* < 0.001) and a significant positive effect on work withdrawal behavior (*β* = 0.207, *p* < 0.001). Work-life balance, in turn, exerted a significant negative influence on work withdrawal behavior (*β* = −0.439, *p* < 0.001).

### Mediation analysis of perceived stress, work-life balance, and work withdrawal behavior among participants

The mediation effect was tested using the bootstrap method with 5,000 resamples to assess significance and calculate 95% confidence intervals. The results (Table 4) indicated a total effect of 0.310. The direct effect was 0.207, accounting for 66.77% of the total effect. The indirect effect was 0.103, representing 33.23% of the total effect. Therefore, work-life balance plays a significant mediating role between perceived stress and work withdrawal behavior.

**Table 4:** Mediation analysis of perceived stress, work-life balance, and work withdrawal behavior among participants.

Items	Standardized effect	Standard error	*p*	Bootstrap 95% CI	Effect proportion
Indirect effect	0.103	0.019	<0.001	(0.067, 0.143)	33.23%
Direct effect	0.207	0.030	<0.001	(0.147, 0.266)	66.77%
Total effect	0.310	0.027	<0.001	(0.257, 0.362)	100%

## Discussion

This study provides an in-depth examination of the complex relationships among perceived stress, work-life balance, and work withdrawal behavior in ICU nurses within the Chinese context. The contribution of this study lies in applying Conservation of Resources (COR) theory to the extreme stress environment of the ICU, revealing how perceived stress influences withdrawal behavior both directly and indirectly through the erosion of work-life balance.

This study found a significant positive correlation between perceived stress and work withdrawal behavior among ICU nurses, supporting Hypothesis 1. This result is consistent with existing literature (4, 38); however, our findings further suggest that in the unique environment of the ICU, which is characterized by frequent life-and-death decisions and high-load responsibilities (39, 40), the impact of perceived stress on withdrawal behavior may be particularly pronounced (40, 41). From the perspective of COR theory, persistent high stress depletes nurses’ personal resources, such as time, energy, and social support (42). When the threat of resource loss persists, individuals may adopt maladaptive work withdrawal behavior (e.g., absenteeism, reduced efficiency, diminished empathy toward patients) as a means of preserving remaining resources (42). Notably, the stress-to-withdrawal pathway is not linear or inevitable—individual differences (e.g., psychological resilience, coping styles) and organizational factors (e.g., colleague support, managerial fairness) may moderate this relationship (6). Thus, while our findings are consistent with prior research, they extend the literature by documenting the stress-withdrawal link specifically in the high-intensity ICU context.

This study validated Hypothesis 2, confirming a statistically significant negative correlation between work-life balance and work withdrawal behavior among ICU nurses (*β* = −0.439, *p* < 0.001). This empirical finding is consistent with previous research conducted on general nurse populations (4, 15). As outlined in COR theory, work-life balance can be viewed as a key personal resource. It can therefore be inferred that when nurses’ work-life balance is impaired, they may experience a net loss of time, energy, and opportunities for recovery, leading them to engage in behaviors such as absenteeism, lateness, or emotional disengagement in order to preserve remaining resources (5, 43). It should be noted, however, that the above mechanism is a theory-driven interpretation, and its causal pathways await direct testing in future research. Furthermore, the observed effect size is modest, indicating that work-life balance is only one of many factors influencing work withdrawal behavior, rather than a “necessary and sufficient condition” for it. Previous studies have suggested that variables such as perceived organizational support, professional commitment, or individual coping styles may play moderating or mediating roles in this relationship (44, 45). The results of this study only preliminarily suggest that improving ICU nurses’ work-life balance might help reduce their work withdrawal behavior; however, this inference remains exploratory and should be interpreted with caution.

This study further revealed that work-life balance partially mediates the relationship between perceived stress and work withdrawal behavior, accounting for 33.23% of the total effect (i.e., the indirect effect explained about one-third of the total variance), thereby supporting Hypothesis 3. To our knowledge, this study provides quantitative evidence of a resource loss pathway in which perceived stress triggers work withdrawal behavior by impairing work-life balance among ICU nurses. Consistent with COR theory (5), when ICU nurses are chronically exposed to high-intensity stressors (e.g., life-and-death decisions, excessive shift loads), their ability to maintain a balance between work and non-work domains is among the first resources to be eroded (16); the decline in work-life balance further deprives individuals of the time and psychological resources needed for recovery and coping, ultimately manifesting as withdrawal behavior such as absenteeism and turnover intention (4, 46). Of note, the mediation effect explained only about one-third of the total effect, indicating that other parallel mediators (e.g., job burnout, perceived organizational support, self-efficacy) are not captured in our model (47). Hence, work-life balance should not be viewed as the sole mediator between perceived stress and withdrawal behavior.

Based on the above empirical findings, we offer the following exploratory management considerations. At the individual level, it may be beneficial to consider incorporating the Perceived Stress Scale into annual mental health assessments for ICU nurses and to provide low-threshold psychological counseling for those with high scores. At the organizational level, possible strategies include implementing a combination of fixed-cycle rotation and self-scheduling (ensuring that consecutive working hours do not exceed 8 h and that there are two full days of rest every 2 weeks), establishing an emergency backup staffing pool to reduce unscheduled overtime, and creating a “recovery corner” within the ICU along with peer support programs. At the cultural level, developing family-friendly policies (e.g., flexible shift adjustments in emergency situations) and providing “resource-conserving leadership” training for head nurses—such as identifying signs of resource depletion, offering flexible shift arrangements, and establishing transparent and fair performance feedback mechanisms—could be considered. It is important to emphasize that these suggestions remain preliminary and theory-driven; the effectiveness of any such interventions has not been directly tested in the current study.

We call for future studies to employ more rigorous designs (e.g., longitudinal cross-lagged panel models or randomized controlled trials) to explicitly test whether interventions aimed at improving work-life balance can significantly reduce the indirect effect of perceived stress on withdrawal behavior, and to explore potential moderators (e.g., management trust, implementation of family-friendly policies) to guide targeted interventions. Until higher-level evidence is available, healthcare administrators should implement the suggested measures with caution, conducting small-scale pilot tests adapted to their specific institutional contexts rather than adopting them wholesale.

Several limitations should be considered in this study. First, the cross-sectional design precludes causal inferences among the variables; longitudinal designs are recommended in future research to further examine causal relationships. Second, although the sample size was substantial, participants were mainly ICU nurses from 16 provinces and municipalities in China, which may limit the generalizability of the findings due to regional and hospital-type variations. Expanding the sampling scope in future studies is therefore suggested. Finally, the relationship between perceived stress and work withdrawal behavior might also be influenced by other mediating or moderating variables, such as job burnout or social support. Future studies could develop more comprehensive models to further explore these mechanisms.

## Conclusion

This study aimed to examine the relationships between perceived stress, work-life balance, and work withdrawal behavior among ICU nurses. The results indicate a significant association between perceived stress and work withdrawal behavior, with work-life balance playing a crucial mediating role in this relationship. These findings contribute to a deeper understanding of the underlying mechanisms of work withdrawal behavior in ICU nurses and provide a theoretical basis for developing effective interventions to improve nurses’ occupational health and wellbeing. Enhancing nurses’ work-life balance may help alleviate perceived stress and further reduce the incidence of work withdrawal behavior, thereby positively influencing the quality and stability of healthcare delivery.

## Data Availability

The original contributions presented in the study are included in the article/Supplementary material, further inquiries can be directed to the corresponding author.
