# Supplementary material for: Work-life balance as a mediator between perceived stress and work withdrawal behavior among critical care nurses in China: a multicenter cross-sectional study
Source: Front Public Health. 2026 May 8;14:1776976. doi: 10.3389/fpubh.2026.1776976 (PMC13194609; doi:10.3389/fpubh.2026.1776976)

Supplementary Material

1 Supplementary Tables

Table1: General Characteristics of the Participants

Variable	n (%)	Work Withdrawal Behavior (Mean±SD)	Statistic	*P*
Total	1279 (100)	20.374 ± 8.235		
Hospital Nature			t=-1.510	0.142
State hospital	1249 (97.65)	20.287 ± 8.059		
Private hospital	30 (2.35)	24.000 ± 13.409		
Hospital level			F = 0.026	0.975
Tertiary hospital	1134 (88.66)	20.368 ± 8.371		
Secondary hospital	134 (10.48)	20.463 ± 7.064		
Primary hospital	11 (0.86)	19.909 ± 7.943		
Department			F = 2.551	0.078
General ICU	1110 (86.79)	20.570 ± 8.339		
Surgical ICU	91 (7.12)	19.407 ± 8.245		
Medical ICU	78 (6.10)	18.705 ± 6.335		
ICU Work Duration			F = 5.334	0.001
1-5	506 (39.56)	19.257 ± 7.837		
6-10	348 (27.21)	21.218 ± 8.001		
11-20	384 (30.02)	21.086 ± 8.694		
≥21	41 (3.21)	20.317 ± 9.248		
Position			t=-0.246	0.806
Clinical	1135 (88.74)	20.359 ± 8.494		
Administrative	144 (11.26)	20.493 ± 5.829		
Professional Title			F = 4.932	0.007
Primary	710 (55.51)	19.808 ± 8.457		
Intermediate	502 (39.25)	20.888 ± 7.921		
Senior	67 (5.24)	22.507 ± 7.640		
Education			F = 0.082	0.922
Associate degree and below	235 (18.37)	20.187 ± 9.109		
Bachelor’s degree	1002 (78.34)	20.409 ± 8.075		
Master’s degree and above	42 (3.28)	20.571 ± 6.936		
Age			F = 6.770	0.001
20-29	476 (37.22)	19.294 ± 7.682		
30-39	678 (53.01)	21.091 ± 8.586		
≥40	125 (9.77)	20.592 ± 7.941		
Service Years			F = 8.607	<.001

Variable	n (%)	Work Withdrawal Behavior (Mean±SD)	Statistic	*P*
1-5	407 (31.82)	18.985 ± 7.508		
6-10	334 (26.11)	20.949 ± 8.161		
≥11	538 (42.06)	21.067 ± 8.679		
Gender			t = 1.692	0.091
Male	217 (16.97)	21.235 ± 8.354		
Female	1062 (83.03)	20.198 ± 8.203		
Marital Status			F = 10.252	<.001
Married	828 (64.74)	21.087 ± 8.676		
Unmarried	422 (33.00)	18.900 ± 7.071		
Divorced or widowed	29 (2.27)	21.448 ± 8.454		
Night Shift			t = 1.373	0.170
No	162 (12.67)	21.204 ± 7.353		
yes	1117 (87.33)	20.253 ± 8.351		
Monthly Income			F = 3.211	0.022
≤5000	259 (20.25)	20.336 ± 9.226		
5001-8000	539 (42.14)	20.781 ± 8.572		
8001-11000	287 (22.44)	20.749 ± 8.078		
＞11000	194 (15.17)	18.737 ± 5.512		
Presence Of Chronic Disease			t = -3.734	<.001
No	1068 (83.50)	19.993 ± 7.934		
Yes	211 (16.50)	22.299 ± 9.403		

SD: standard deviation，t: t-test, F: ANOVA, ICU: Intensive Care Unit

Table2: Scores on Work-Life Balance, Perceived Stress, and Work Withdrawal Behavior of the Participants

Variables	Score（Mean ± SD）	Item score（Mean ± SD）
Work Life Balance Scale	70.69±18.74	4.16±1.10
Time Balance	23.60±7.33	3.93±1.22
Involvement Balance	21.48±5.68	4.30±1.14
Satisfaction Balance	25.61±6.96	4.27±1.16
Work Withdrawal Behavior Scale	20.37±8.24	1.70±0.69
Psychological Withdrawal	14.97±5.84	1.87±0.73
Behavioral Withdrawal	5.40±2.92	1.35±0.73
Perceived Stress Scale	33.03±9.14	3.30±0.91
Negative Stress Perception	19.77±6.19	3.29±1.03
Positive Stress Perception	13.27±4.06	3.32±1.02

Table3: Correlation Analysis of Work-Life Balance, Perceived Stress, and Work Withdrawal Behavior among the Participants

Correlations	Perceived Stress Scale	Work Life Balance Scale	Work Withdrawal Behavior Scale
Perceived Stress Scale	1		
Work Life Balance Scale	-0.203**	1	
Work Withdrawal Behavior Scale	0.261**	-0.457**	1

**，P＜0.01

***Table4** Mediation Analysis of Perceived Stress, Work-Life Balance, and Work Withdrawal Behavior among Participants*

Items	Standardized [illegible]	Standard	P	Bootstrap 95% CI	Effect Proportion
Indirect effect	0.103	0.019	＜0.001	(0.067,0.143)	33.23%
Direct effect	0.207	0.030	＜0.001	(0.147,0.266)	66.77%
Total effect	0.310	0.027	＜0.001	(0.257,0.362)	100%

2 Supplementary **Figure**

Figure 1：Structural Equation Modeling for Perceived Stress, Work-life Balance, and Work Withdrawal Behavior Among Participants

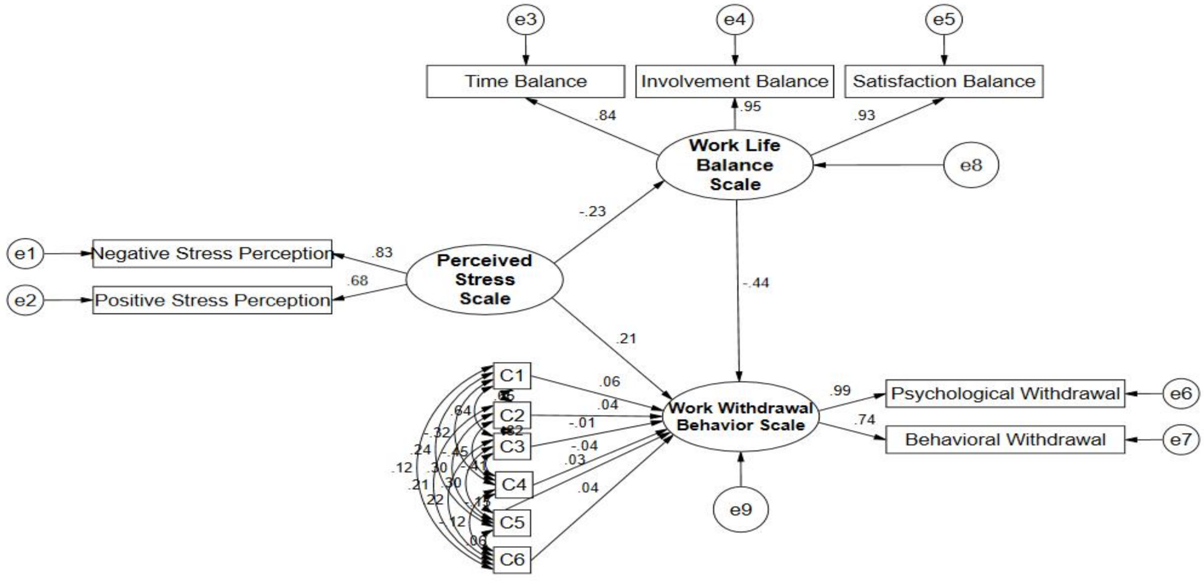

Supplement: Supplementary file 1 [file Data_Sheet_1.pdf]
